# Mining Eco-Efficiency Measurement and Driving Factors Identification Based on Meta-US-SBM in Guangxi Province, China

**DOI:** 10.3390/ijerph18105397

**Published:** 2021-05-18

**Authors:** Yonglin Li, Zhili Zuo, Deyi Xu, Yi Wei

**Affiliations:** 1 School of Economics and Management, China University of Geosciences, Wuhan 430074, China; lyl2018@cug.edu.cn (Y.L.); zhilizuo.eva@gmail.com (Z.Z.); yige88888@163.com (Y.W.); 2 Research Center of Resource and Environment Economics, Mineral Resource Strategy and Policy Research Center, China University of Geosciences, Wuhan 430074, China

**Keywords:** mining eco-efficiency, Meta-US-SBM, standard deviation ellipse model, GeoDetector, Tobit Model, Guangxi

## Abstract

The mining industry is one of the pillar industries of Guangxi’s economic and social development. The output value of mining and related industries accounts for 27% of the whole district’s total industrial output value. Therefore, the mining eco-efficiency measurement in Guangxi can be of great significance for the sustainable development of Guangxi’s mining industry. This study adopted Meta-US-SBM to measure the mining eco-efficiency in Guangxi from 2008 to 2018, including economic efficiency, resource efficiency, and environmental efficiency. It used the standard deviation ellipse model to simulate the migration trend of four efficiencies in Guangxi and used GeoDetector and Tobit models to explore the internal and external factors that affect the mining eco-efficiency. The four efficiencies in Guangxi show large temporal and spatial heterogeneity, and the internal and external factors that affect the mining eco-efficiency are different. The following conclusions can be drawn. (1) Environmental efficiency and mining eco-efficiency are improving, while economic efficiency and resource efficiency are deteriorating. Cities bordering other provinces have a significantly better mining eco-efficiency than non-bordering cities. (2) The development center in Guangxi has migrated to the Beibu Gulf Economic Zone. (3) Natural resources index and mining economic scale have a great impact on the mining eco-efficiency, and with the increase of the mining economic scale, the mining eco-efficiency showed a typical “U-shaped” curve. Finally, this study put forward corresponding policy recommendations to improve the mining eco-efficiency in Guangxi from four aspects: opening-up, technological progress, regional coordination, and government control.

## 1. Introduction

China has achieved rapid economic development over the past ten years, while the over-utilization of resources and high emissions of pollutants have seriously hindered its sustainable development. Although China is committed to establishing an environment-friendly and resource-conserving society, it has not yet transformed from a high-emission growth mode to a sustainable mode [1]. It is estimated that China’s annual economic losses due to environmental pollution and ecological damage account for 6% of its gross domestic product (GDP) [2]. China still needs to make more efforts on the road to achieving sustainable development. As Burton [3] pointed out that sustainable development refers to development that meets the present needs without compromising the ability of future generations to meet their own needs. Eco-efficiency has been proposed as a route to promote such a transformation [4].

Eco-efficiency was derived from the concept of “environmental efficiency” in the 1970s by Freeman et al. [5], and then Schaltegger and Sturm [6] introduced it as the ratio of the economic value created to the environmental impact generated. In 1992, the World Business Council disclosed the term as the index of economic and environmental efficiency, namely as a management strategy that links financial and environmental performance to create more value with less ecological impact [7]. Later, it was defined by Organization for Economic Co-operation and Development (OECD) [8] as “the efficiency with which ecological resources are used to meet human needs”, providing firms, industries, or economies with the ability to produce goods and services with less impact on the environment while consuming fewer natural resources [9]. In other words, eco-efficiency increases when the impact of economic production on ecosystem services is reduced [10], when the increase of the economic production value corresponds to a decrease of environmental impacts [11].

As an instrument for sustainability analysis, eco-efficiency has received significant attention in the sustainable development literature and has become the topic of a growing body of studies. Eco-efficiency can be measured as the ratio between the (added) value of what has been produced and the (added) environmental impacts of the product or service [12]. However, it is insufficient to research eco-efficiency using a single indicator [13]. There are four main methods of determining eco-efficiency, including the indicator system method [14], the ratio approach [15], life-cycle assessment [16], and the frontier approach [17], among which Data Envelopment Analysis (DEA) is the most frequently applied. DEA is a well-known frontier approach that calculates the input–output efficiency of decision-making units using a programming solver. It was first proposed by Charnes and Cooper [18], and then, Färe et al. [19] integrated the DEA method with the Malmquist index to examine the effects of dynamic changes and resource utilization efficiency. Tone [20] further proposed a non-radial slacks-based measure (SBM) model based on relaxation measurements and incorporate undesirable outputs into the DEA evaluation.

Eco-efficiency has been applied from many perspectives, such as the macro-economic [21], the meso-economic [22], and the micro-economic levels [23]. Liu et al. [24] considered the provincial panel data in China during 1978–2017 to measure the agricultural eco-efficiency by the super-efficiency slacks-based measure (super-SBM) model. Peng et al. [25] created a comprehensive evaluation index system, including undesirable outputs, and adopted SBM to analyze the characteristics and evolution of eco-efficiency at an individual tourism destination. Zhang et al. [12] used real data of 30 provinces in China and employed an empirical study to illustrate the pattern of regional industrial systems’ eco-efficiency. Hu and Liu [26] used Australian construction industry data from 1990 to 2013 to assess eco-efficiency based on directly and systematically dealing with the slacks of reducing resource consumption and minimizing environmental impacts, together with adding production value. Li and Hu [27] computed the ecological total-factor energy efficiency of 30 provinces in China for 2005–2009 through the SBM with undesirable outputs. Wang et al. [28] investigated the eco-efficiency trends of Shandong Province’s Pulp and Paper Industry from 2001 to 2008 in three fields related to water efficiency, energy efficiency, and environment efficiency. They used a “de-linking” and “re-linking” tool to attain a further evaluation. Table 1 shows the use of DEA methodology over the years for assessing the eco-efficiency.

Currently, most of the research on eco-efficiency focuses on urban agglomerations [39] at the national level [36] or provincial level [28], and the research from prefecture-level cities perspective is scarce. Ignoring the differences between cities will affect the applicability of the research results due to the regional heterogeneity. The existing eco-efficiency is mainly analyzed from tourism, construction, agriculture, industry, etc., and there are few studies on the mining eco-efficiency. Hence, this study is of great theoretical and practical importance, as it will contribute towards the analysis of the mining eco-efficiency. The research aims to analyze the temporal and spatial variation of Guangxi’s mining eco-efficiency and the internal and the external driving factors, which is of great significance to improve its mining eco-efficiency and the sustainable development of mining in Guangxi.

## 2. Methodology and Data Source

### 2.1. Study Site

**Figure 1:**
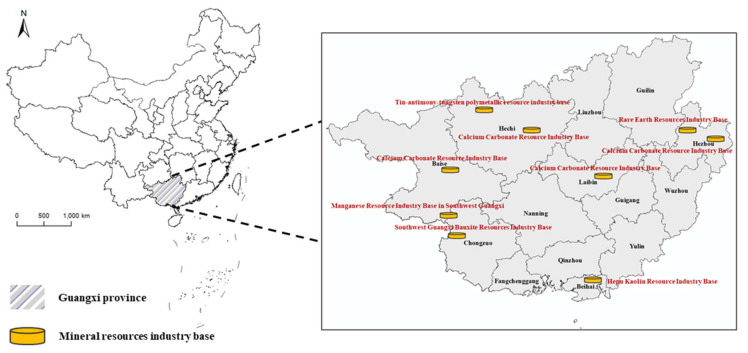
Location of the study area.

Guangxi is located on the central and southern regions (104°26′–112°04′ E, 20°54′–26°24′ N), as shown in Figure 1. There are 14 cities in Guangxi, covering a total area of over 236,700 km^2^, with occupies 2.47% of China’s land area. The overall economy of Guangxi has developed rapidly. Guangxi is rich in mineral resources, with a wide variety of types and large reserves, especially non-ferrous metals such as aluminum and tin. It is one of the ten key non-ferrous metal production areas in China. There are currently 145 kinds of minerals such as manganese, aluminum, tin, iron, arsenic, bentonite, vanadium, tungsten, indium, lead, zinc, and antimony silver that have been discovered in the territory, and 97 kinds of mineral reserves have been proven. According to The Overall Planning of Mineral Resources in Guangxi (2016–2020), the output value of mining and related energy and raw material processing and manufacturing industries was 673.8 billion yuan, accounting for 31% of the region’s total industrial output value. There are obvious regional differences in the distribution of mineral resources, mainly in Hechi, Baise, Chongzuo, etc. Most (82%) of manganese reserves are concentrated in Chongzuo and Baise, 96% of aluminum reserves are concentrated in Baise, 67% of tin reserves are concentrated in Hechi, and over 85% of rare earth reserves are concentrated in Yulin, Hezhou, and Guigang; 79% of the barite resource reserves are concentrated in Liuzhou and Laibin, 88% of the kaolin resource reserves are concentrated in Beihai, and 88% of the coal resource reserves are concentrated in Baise and Laibin. Part of the mineral reserves in Guangxi are even at the forefront of the world, so Guangxi is also known as the “hometown of non-ferrous metals.” [40] Thus, it is essential to study the sustainability of mining, the results of which may accelerate the construction of a major mining province and provide policy recommendations for promoting the sustainable economic and social development [41].

### 2.2. Definition of the Composite Mining Eco-Efficiency

Eco-efficiency refers to creating more goods and services while having less impact on the environment and less consumption of natural resources. It involves all aspects of the economy and society and is a complex and multi-dimensional problem [13]. This study constructs a three-dimensional analysis framework to reveal the relationship between ecological efficiency, environmental efficiency, resource efficiency, and economic efficiency. These four types of efficiencies are all based on total factor productivity. In order to maximize it, economic growth, resource assumptions, and environmental pollutants cannot be fixed, indicating that non-orientation should be more appropriate when measured eco-efficiency [30]. The meaning of eco-efficiency in this research: maximization of economic output, minimization of environmental pollution, and minimization of energy consumption.

Economic efficiency refers to the efficiency of a decision-making unit to obtain economic output at a given time for various resource inputs and undesired output, and other factors, reflecting its relative potential to maximize economic output. Therefore, this study adopted an output-oriented model and ensured that the slack of other output variables is zero. In order to identify frontier decision-making units and ensure inter-temporal comparability, a super-efficiency model is required. Considering non-radial variation and heterogeneity, SBM models and meta-frontiers need to be adopted [42].

Environmental efficiency refers to the efficiency of a decision-making unit in minimizing the output of environmental pollutants in production, reflecting its relative potential to obtain a given economic output at a minimum environmental cost when other factors such as various resource inputs and economic output are established. This study adopted an output-oriented model to measure environmental efficiency [30,43].

Resource efficiency refers to the relative efficiency of a decision-making unit’s resource utilization in production when other input variables and output variables are established, reflecting its relative potential to maximize resource utilization. Therefore, this study adopted an input-oriented model, ensured that the slack of the output variable is zero, and used the slack variable corresponding to the energy input variable to measure the optimal resources input; then, it used the ratio of the optimal resources input to the actual usage to measure resource efficiency [44].

**Figure 2:**
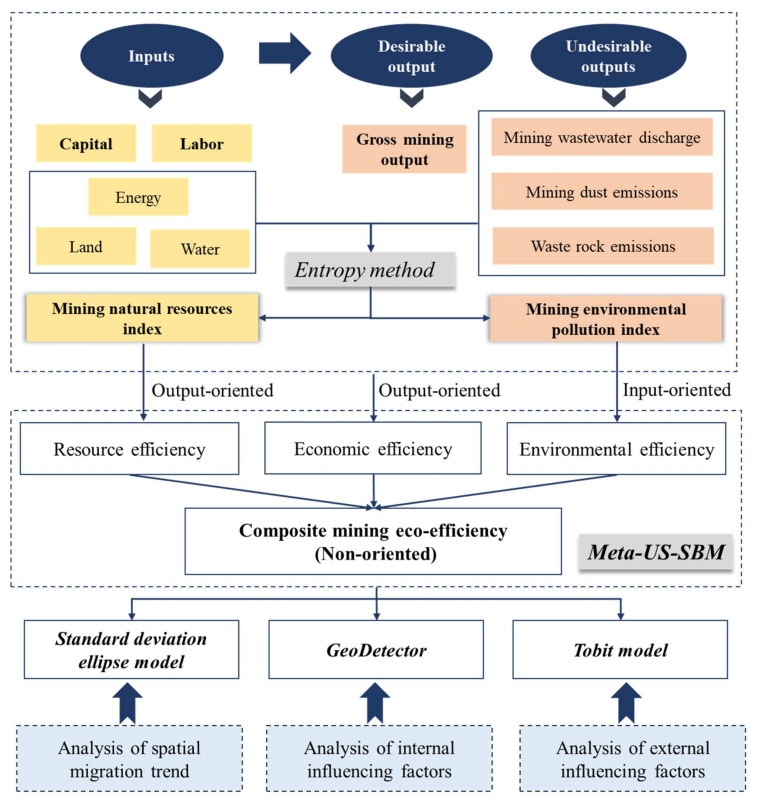
Research framework.

The research framework includes three parts: (1) First, it constructed a measurement model of mining eco-efficiency based on Meta Undesirable Meta-US-SBM and analyzed the mining eco-efficiency development trends from 2008 to 2018, which enriched the field of eco-efficiency. (2) Then, it analyzed the heterogeneity and spatial effects of Guangxi’s mining eco-efficiency from both temporal and spatial dimensions, which demonstrated comprehensively the mining eco-efficiency in Guangxi. (3) Lastly, it adopted the GeoDetector and Tobit model to study the internal and external influencing factors of mining eco-efficiency, which provided an exhaustive analysis of its driving factors. The research framework is shown in Figure 2.

### 2.3. Meta-US-SBM to Measure Mining Eco-Efficiency

Considering the regional heterogeneity in Guangxi, a metafrontier analysis is necessary [45], which include two steps: (1) classifying the prefectural-level cities into different groups according to their characteristics (e.g., geographical location, economic development level, population, income levels, etc.) and estimating a production frontier for each group, and then (2) estimating the metafrontier by enveloping the group-specific frontiers [46]. Compared with the traditional DEA model, Meta-US-SBM has a superior ability in identifying fully the inter-temporal comparability of DMUs [32].

Assume that the number of observed DMUs is N and that they can be divided into H groups according to heterogeneities, each group containing $N_{h}$ DMUs and $\sum_{h=1}^{H}N_{h}=N$. Then, each DMU uses inputs $x=\left[x_{1},x_{2},\ldots ,x_{M}\right]\in R_{+}^{M}$ to produce desirable outputs $y=\left[y_{1},y_{2},\ldots ,y_{R}\right]\in R_{+}^{R}$ and undesirable outputs $b=\left[b_{1},b_{2},\ldots ,b_{J}\right]\in R_{+}^{J}$. The frontier production technology of group  h can be expressed as follows [43]:

(1) $$p^{meta}=\left\{\left(x,y,b\right):\overset{H}{\underset{h=1}{\sum }}\overset{N_{h}}{\underset{n=1}{\sum }}\xi_{n}^{h}x_{n}^{h}\leq x^{h};\overset{H}{\underset{h=1}{\sum }}\overset{N_{h}}{\underset{n=1}{\sum }}\xi_{n}^{h}y_{n}^{h}\leq y^{h};\;\overset{H}{\underset{h=1}{\sum }}\overset{N_{h}}{\underset{n=1}{\sum }}\xi_{n}^{h}b_{n}^{h}\leq b^{h};\;\xi_{n}^{h}\geq 0;n=1,2,\ldots ,N_{h};h=1,2,\ldots ,H\right\}$$

where $p^{meta}=\left\{P^{1}\cup P^{2}\cup \ldots \cup P^{H}\right\}$, and $\xi_{n}^{h}$ is the weight for $n_{th}\;DMU$ in the $h_{th}$ group under the meta-frontier.

The optimal solution of the proposed non-oriented Meta-US-SBM model can be estimated as follows:

$$\rho_{ko}{}^{Meta*}=min\frac{1+\frac{1}{M}\sum_{m=1}^{M}\frac{s_{mko}^{x}}{x_{mko}}}{1-\frac{1}{R+J}\left(\sum_{r=1}^{R}\frac{s_{rko}^{y}}{y_{rko}}+\sum_{j=1}^{J}\frac{s_{jko}^{b}}{b_{jko}}\right)}$$

$$s.t.\;x_{mko}-\sum_{h=1}^{H}\sum_{n=1,n\neq 0\;\;if\;\;h=k}^{N_{h}}\xi_{n}^{h}x_{mhn}+s_{mko}^{x}\geq 0$$

$$\sum_{h=1}^{H}\sum_{n=1,n\neq 0\;\;\;if\;\;h=k}^{N_{h}}\xi_{n}^{h}y_{rhn}-y_{rko}+s_{rko}^{y}\geq 0$$

$$b_{jko}-\sum_{h=1}^{H}\sum_{n=1,n\neq 0\;\;if\;\;h=k}^{N_{h}}\xi_{n}^{h}b_{jhn}+s_{jko}^{b}\geq 0$$

$$1-\frac{1}{R+J}\left(\sum_{r=1}^{R}\frac{s_{rko}^{y}}{y_{rko}}+\sum_{j=1}^{J}\frac{s_{jko}^{b}}{b_{jko}}\right)\geq \varepsilon$$

(2) $$\xi_{n}^{h},\;s^{x},s^{y},s^{b}\geq 0;\;m=1,2,\ldots ,M;r=1,2,\ldots ,R;j=1,2,\ldots ,J$$

where $s^{x},s^{y},\;s^{b}$ are the slacks of inputs, desirable outputs, and undesirable outputs, respectively. $\rho_{ko}{}^{Meta*}$ is the measured meta-frontier efficiency of the $o_{th}\;DMU$ in the $k_{th}$ group. Under the assumption of variable returns of scale, $\sum_{h=1}^{H}\sum_{n=1,n\neq 0\;if\;h=k}^{N_{h}}\xi_{n}^{h}=1$ is necessary.

The definitions of the composite mining eco-efficiency indicators, including eco-efficiency and its three sub-efficiencies, are clarified in Section 2.2. Eco-efficiency is evaluated by Equation (2), the non-oriented Meta-US-SBM model. As mining eco-efficiency reflects the comprehensive degree of coordination between economy, resource, and environment [47], reducing resource consumption as well as undesirable outputs and raising desirable production should be taken into account simultaneously. Therefore, the slacks of both inputs and outputs need to be changed according to the specific situation [30].

Economic efficiency means that the input and undesired output of a DMU are assumed to be the same as others, so the slacks, except for desirable outputs, equal zero. This is consistent with the connotation of the output-oriented efficiency measurement. The specific measures are formulated as follows:

$$\delta_{ko}{}^{Meta*}=min\frac{1}{1-\frac{1}{R}\sum_{r=1}^{R}\frac{s_{rko}^{y}}{y_{rko}}}$$

$$s.t.\;x_{mko}-\sum_{h=1}^{H}\sum_{n=1,n\neq 0\;\;\;if\;\;h=k}^{N_{h}}\xi_{n}^{h}x_{mhn}\geq 0$$

$$\sum_{h=1}^{H}\sum_{n=1,n\neq 0\;\;\;if\;\;h=k}^{N_{h}}\xi_{n}^{h}y_{rhn}-y_{rko}+s_{rko}^{y}\geq 0$$

$$b_{jko}-\sum_{h=1}^{H}\sum_{n=1,n\neq 0\;\;\;if\;\;h=k}^{N_{h}}\xi_{n}^{h}b_{jhn}\geq 0$$

(3) $$\xi_{n}^{h},\;s_{rko}^{y}\geq 0;m=1,2,\ldots ,M;r=1,2,\ldots ,R;j=1,2,\ldots ,J.$$

When assessing environmental efficiency, the slacks of resource inputs and economic production are assumed to be zero. It can be calculated using the following output-oriented model:

$$\theta_{ko}{}^{Meta*}=min\frac{1}{1-\frac{1}{J}\sum_{r=1}^{R}\frac{s_{jko}^{b}}{b_{jko}}}$$

$$s.t.\;x_{mko}-\sum_{h=1}^{H}\sum_{n=1,n\neq 0\;\;\;if\;\;h=k}^{N_{h}}\xi_{n}^{h}x_{mhn}\geq 0$$

$$\sum_{h=1}^{H}\sum_{n=1,n\neq 0\;\;\;if\;\;h=k}^{N_{h}}\xi_{n}^{h}y_{rhn}-y_{rko}\geq 0$$

$$b_{jko}-\sum_{h=1}^{H}\sum_{n=1,n\neq 0\;\;\;if\;\;h=k}^{N_{h}}\xi_{n}^{h}b_{jhn}+s_{jko}^{b}\geq 0$$

(4) $$\xi_{n}^{h},\;s_{jko}^{b}\geq 0;m=1,2,\ldots ,M;r=1,2,\ldots ,R;j=1,2,\ldots ,J.$$

Generally, scholars use the ratio of target input to actual input as resource efficiency [48]. This study employed the input-oriented Meta-US-SBM approach to measure resource efficiency. It can be defined as follows:

$$\eta_{ko}{}^{Meta*}=\min(1+\frac{1}{M}\sum_{m=1}^{M}\frac{s_{mko}^{x}}{x_{mko}})$$

$$s.t.\;x_{mko}-\sum_{h=1}^{H}\sum_{n=1,n\neq 0\;\;\;if\;\;h=k}^{N_{h}}\xi_{n}^{h}x_{mhn}+s_{mko}^{x}\geq 0$$

$$\sum_{h=1}^{H}\sum_{n=1,n\neq 0\;\;\;if\;\;h=k}^{N_{h}}\xi_{n}^{h}y_{rhn}-y_{rko}\geq 0$$

$$b_{jko}-\sum_{h=1}^{H}\sum_{n=1,n\neq 0\;\;\;if\;\;h=k}^{N_{h}}\xi_{n}^{h}b_{jhn}\geq 0$$

(5) $$\xi_{n}^{h},\;s_{mko}^{x}\geq 0;m=1,2,\ldots ,M;r=1,2,\ldots ,R;j=1,2,\ldots ,J.$$

Then, according to Equation (5), it can measure the efficiency of the $m_{th}$ resource based on the slack scalar $s_{mko}^{x}$ and actual input $x_{mko}$.

(6) $$RE_{ko}{}^{Meta*}=\frac{x_{mko}-s_{mko}^{x}}{x_{mko}}$$

where $x_{mko}-s_{mko}^{x}$ denotes the target volume of input, $x_{mko}$ is the actual input.

### 2.4. GeoDetector

The GeoDetector method is a quantitative technique that determines whether the spatial distribution of a geostatistical variable is consistent with an independent variable [49]. The fundamental theory of the GeoDetector was first proposed by Wang et al. [50] as a method of detecting the risks of neural tube defect diseases. The GeoDetector applies the q value to measure the heterogeneity and autocorrelation of the dependent variable quantitatively and detects the association between the dependent variable and its influencing factors [51].

(7) $$q=1-\frac{\sum_{h=1}^{L}N_{h}\sigma_{h}^{2}}{N\sigma^{2}}$$

where N is the number of samples in the study area; $N_{h}$ is the number of samples in zone category h of factor X; $\sigma^{2}$ is the total variance of γ in the study area; $\sigma_{h}^{2}$ is the variance of γ within category  h of factor X; and L is the number of categories of factor X. $\sum_{h=1}^{L}N_{h}\sigma_{h}^{2}$ is within the sum of variances, and $N\sigma^{2}$ is the total sum of variances. The greater the value of q, the more factor X explains γ [52].

### 2.5. Tobit Model

Tobin [53] proposed the Tobit regression model and mainly addressed the construction problem of limited or truncated dependent variables [54]. The mining eco-efficiency measured by the Meta-US-SBM DEA model is affected by many factors, which include not only the input and output indicators but also some other external factors [55]. This study set the mining eco-efficiency of 14 prefecture-level cities in Guangxi Province as the dependent variable and selected external factors such as the mining economic scale, foreign direct investment, technology innovation, and environmental regulations as independent variables to establish the Tobit model. This model was expressed as follows, and Tobit regression analysis is done by STATA^®^ (StataCorp LLC, Texas, TX, USA).

(8) $$ME_{it}^{*}=\beta x_{it}+\varepsilon_{it},\;y_{it}=\left\{\begin{array}{c} y_{it}^{*},y_{it}^{*}\geq 0\;\;\\ 0,y_{it}^{*}\leq 0\; \end{array}\right.\;i=1,\ldots ,N\;and\;t=1,\ldots ,T,\;\varepsilon_{it}\sim N\left(0,\sigma^{2}\right)$$

where i denotes the 14 prefectural cities in Guangxi, while t represents different years, $x_{it}$ denotes independent variables, while β is a regression parameter, and $\varepsilon_{it}$ is the disturbance term. The selected external influencing factors are described in detail as follows:

(1) Mining economic scale (MES) is represented by the ratio of mining gross output divided by GDP. A higher level of mining economic development in the region means that the ability to gather talent is higher and the ability to absorb technology is stronger, so the ability to promote mining eco-efficiency is also stronger.

(2) Foreign direct investment (FDI) is represented by the portion invested by foreign capital in gross industrial output value. Technological progress has had a profound impact on the environmental results of mining economic activities, and it is also affected by the degree of openness to the outside world [56]. Since the foreign direct investment (FDI) data of the extractive industries in the region cannot be obtained, this paper calculates the ratio of the regional extractive industry output value to the regional GDP and then multiplies it by the FDI of the Chinese extractive industry to replace [57].

(3) Technology innovation (TI) is represented by the ratio of the number of R&D personnel to the total number of employees. The level of technological innovation can reflect the development potential of an industry [58].

(4) Environmental regulation (ER) is represented as the total investment ratio in mining environmental rehabilitation to total investments. Reasonable environmental regulations can stimulate the enthusiasm of enterprises for innovation [59], which can improve the enterprise’s resource optimization level, production efficiency, environmental performance, and technological innovation level [60].

### 2.6. Variables and Data Source

Within the process of production, input and output are the fundamental factors. This study has selected indicators that are all specific indicators that reflect the input and output of the mining industry. Among them, capital, labor, and energy were chosen as the three classic inputs [37], plus two other important inputs: land and water [38]. Then, gross mining output was regarded as a proxy of the desirable output. Mining wastewater discharge, mining dust emissions, and waste rock emissions are identified with the undesirable outputs [32]. In order to simplify the selection of undesired output indicators, this study adopted the entropy method to index them and obtained the mining environmental pollution index as the final undesirable outputs indicator [30].

This study collected data on the above variables for 14 prefecture-level cities in Guangxi over the period from 2008 to 2018. The mining data were derived from Guangxi Statistical Yearbooks (2008–2018), China City Statistical Yearbooks (2008–2018), China Environmental Statistics Yearbook (2008–2018), and China Energy Statistical Yearbooks (2008–2018). Specific indicators are shown in Table 2.

## 3. Results and Discussion

### 3.1. Mining Eco-Efficiency and Spatial Pattern

**Figure 3:**
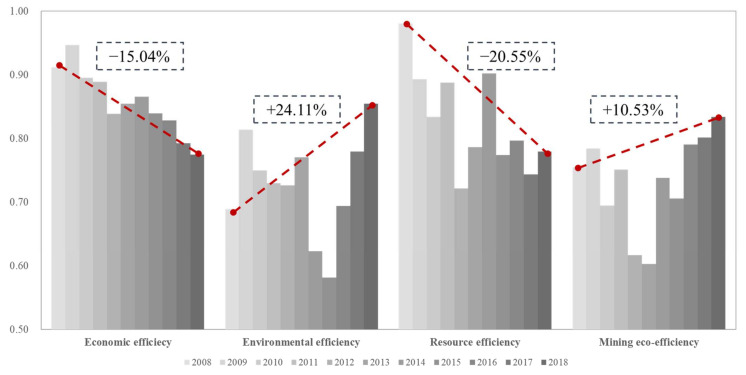
The average value of the eco-efficiency in Guangxi.

The mining eco-efficiency and its three sub-efficiencies in 14 prefecture-level cities in Guangxi from 2008 to 2018 were calculated by the Meta-US-SBM DEA model, and the mining eco-efficiency was discussed from the spatial–temporal dimensions. From Figure 3, it can be concluded that the economic efficiency of Guangxi has shown a downward trend, dropping by 10%; its environmental efficiency and mining eco-efficiency have both shown a “U-shaped” curve, with increasing by 24.11% and 10.53%, respectively; its resource efficiency has shown an “inverted N-shaped curve” and fluctuates a lot. The mining industry is one of the pillar industries of Guangxi’s economic and social development. The output value of mining and related industries accounts for 27% of the total industrial output value of the whole district [61]. In addition, it is also a major industrial province. Such an economic development mode leads to the lowering of the regional economic efficiency, environmental efficiency, resource efficiency, and mining eco-efficiency. However, in 2015, the State Council promulgated the “Overall Plan for the Reform of the Ecological Civilization System”, which proposed to improve the paid use system of mineral resources and the ecological compensation system. It promoted the construction of ecological civilization to an important strategic position, namely, the ecological efficiency and environmental efficiency of the mining industry in Guangxi. Significant improvement has been achieved, which shows that the paid development of mineral resources and the system of mineral resources compensation play an important role in improving the ecological efficiency of China’s mining industry [57].

**Figure 4:**
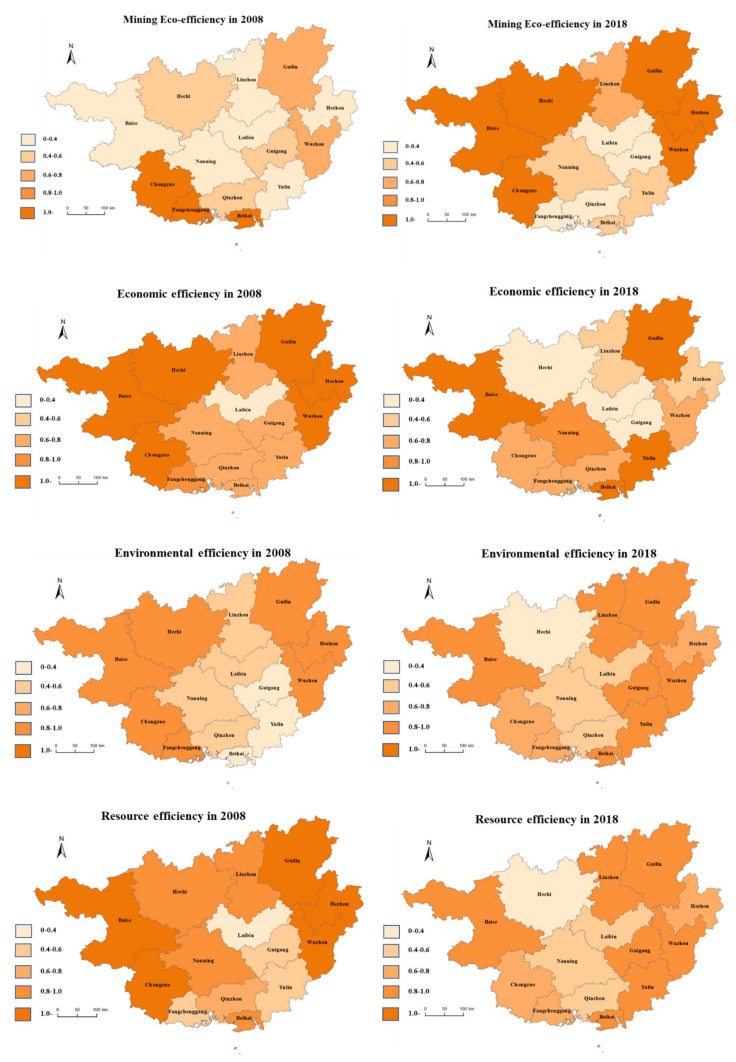
Regional distribution of mining eco-efficiency, economic efficiency, environmental efficiency, and resource efficiency in 2008 and 2018.

From the regional distribution in Figure 4, we can conclude that there exist apparent differences between regions in Guangxi. Overall, economic efficiency and resource efficiency have decreased, while environmental efficiency and mining eco-efficiency have improved. The areas with higher mining eco-efficiency are mainly concentrated in northwest Guangxi and northeast Guangxi. Among them, Hechi, Baise, and Chongzuo belong to the “resource-rich area of western Guangxi”. As an under-developed resource-rich area, the resource-rich area of western Guangxi has focused on developing resource-based industries such as aluminum, manganese, and non-ferrous metals. The resource-based industry and the heavy chemical industry account for a relatively high proportion of all industries in all cities in western Guangxi. Resource-based industries occupy a dominant position in the industrial structure, making the economic growth of the resource-rich areas in western Guangxi a typical resource-consuming and investment-driven type. With the deepening of the construction of ecological civilization, the region has realized the improvement of the mining eco-efficiency by optimizing the industrial layout and promoting the intensive use of land. In addition, the mining eco-efficiency has been improved in bordering cities such as Liuzhou, Guilin, Wuzhou, etc. This benefited from the implementation of the Western Development Strategy. Guangxi relies on its location advantages and resource advantages and actively undertakes manufacturing industries in the eastern region. The transformation and upgrading of the regional industrial structure have improved the regional mining eco-efficiency [62].

From Table 3, we can conclude that different regions show different degrees of economic efficiency, environmental efficiency, resource efficiency, and mining eco-efficiency. In terms of economic efficiency, Beihai, Chongzuo, and Guilin perform better than other regions; Liuzhou, Laibin, and Guigang are relatively poor. In terms of environmental efficiency, Guilin, Wuzhou, and Beihai do better than other regions; Guigang, Hezhou, and Hechi are relatively poor. In terms of resource efficiency, Beihai, Chongzuo, and Hezhou are better than other regions; Guigang, Laibin, and Qinzhou are relatively poor. In terms of mining eco-efficiency, Beihai, Guilin, and Chongzuo are better than other regions; Guigang, Laibin, and Qinzhou are relatively poor.

Among them, the geographical characteristics of Chongzuo give it advantages in the development of the port economy. In 2017, Chongzuo’s GDP grew by 9.3%, ranking second in Guangxi; in 2018, Chongzuo’s GDP grew by 11.3%, the growth rate ranked first in Guangxi; in 2019, Chongzuo’s GDP increased by 8%, with 57 newly added industrial enterprises in scale, and the growth rate ranked second in Guangxi. The positive economic development has steadily improved economic efficiency, and the development of the “port economy” allowed Chongzuo to find new opportunities for economic development [63]. In addition, Guilin and Beihai are popular tourist cities in Guangxi, and their tourism revenue has brought enormous economic development space for the local area, making their economic efficiency rank in the forefront. As the industrial city in Guangxi, Liuzhou has always adhered to the concept of “industry revitalizing the city”. It formed an industrial system with heavy and chemical industries as the mainstay, automobile, metallurgy, and machinery as the three pillar industries, which has promoted Liuzhou’s industrial economic development. However, industrial economic development has also brought about negative characteristics such as environmental degradation and resource consumption. Therefore, although Liuzhou’s economic development is relatively good, its economic efficiency is relatively backward. This also reflects that Liuzhou urgently needs to adjust the industrial structure and use technological innovation to achieve the harmonious development of the “economy–social–environment.”

In summary, we can conclude that the quality of economic efficiency does not depend on the total economic development or economic development level. It focuses more on whether the regional economic development mode has achieved efficient development. Therefore, the improvement of economic efficiency requires an improved coordination of economy, environment, and society. As the largest inland port city in Southwest China, Guigang has outstanding warehousing, ship construction, and machinery manufacturing. However, its urbanization level and urbanization quality rank low in Guangxi Province. In addition, Guigang is the only prefecture-level city in Guangxi without a university. The backwardness of higher education has directly caused the inconsistency of job positions and the quality of workers, which has caused the transformation and upgrading of the industrial structure to be out of touch with economic development. All the above factors make Guigang’s economic efficiency, environmental efficiency, resource efficiency, and mining ecological efficiency rank low [64].

**Figure 5:**
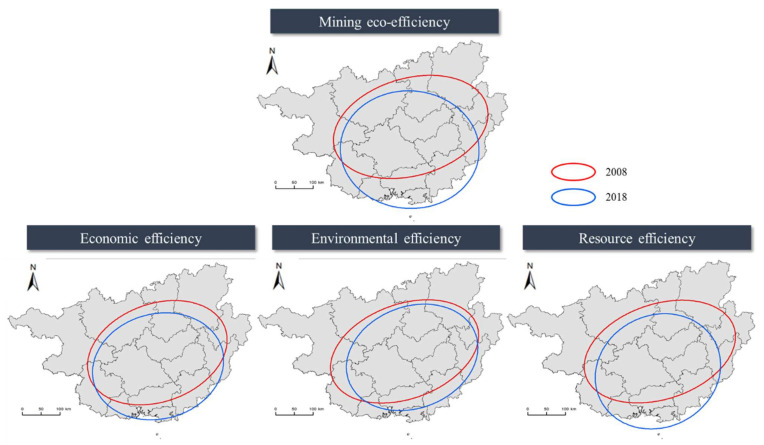
Standard deviational ellipses of mining eco-efficiency in Guangxi.

The SDE model was used to visually express the spatial distributions and dynamic evolutionary processes of efficiencies by ArcGIS, for which the major standard ellipse axis reflected the spatial distribution element ranges. The size of the ellipse reflects the spatial concentration of efficiencies, and the semi-major axis reflects the dominant direction of the efficiencies. The parameter changes of the standard deviation ellipses of regional mining eco-efficiency are shown in Table 4, and the spatial distribution is shown in Figure 5. We can conclude that Guangxi’s mining eco-efficiency, economic efficiency, environmental efficiency, and resource efficiency show significant regional migration trends.

The distribution of the four efficiencies in Guangxi tends to be a circle, reflecting a relatively balanced distribution of efficiencies in Guangxi. With economic development and strategic tilt, regional development disparity is gradually narrowing, leading to a balance in regional efficiency. From 2008 to 2018, the ratios of the long and short axes of the mining eco-efficiency, economic efficiency, environmental efficiency, and resource efficiency increased by 0.563, 0.114, 0.137, and 0.262, respectively. The increase in the long and short axis ratio indicates the expansion of the effective area, indicating the overall trend of improvement. From 2008 to 2018, the rotation gaps in mining eco-efficiency have significant increasing trends. The ellipse was rotating clockwise, and the direction of the ellipse being elongated to the south and east, indicating that the mining eco-efficiency in the south, east, and southeast of the ellipse were faster. The above results show that the development of the center area of mining eco-efficiency, economic efficiency, environmental efficiency, and resource efficiency is gradually shifting to the Beibu Gulf Economic Development Zone. Currently, many developed countries regard ports as the breakthrough point for the development of logistics, promote the development of port industry through the development of port logistics, and radiate the surrounding areas to promote import and export trade, which promotes the development of port logistics. With the implementation of the national strategy of the “Guangxi Beibu Gulf Economic Zone Development Plan” and the establishment of the “China-ASEAN Free Trade Area”, the Guangxi Beibu Gulf Economic Zone has become the fastest-growing region in Southwest China. With the further manifestation of the radiation effect of the Beibu Gulf Economic Zone, the mining eco-efficiency, economic efficiency, environmental efficiency, and resource efficiency have become more balanced [65].

### 3.2. Analysis of the Driving Factors of the Mining Eco-Efficiency

Mining eco-efficiency requires as much economic and social benefits as possible with as little environmental cost as possible to achieve a win–win situation of “economy–society–ecology” [55]. It is believed that the spatial differentiation of mining eco-efficiency in Guangxi is attributable to structural factors and economic and social factors, namely internal and external sources. Therefore, this study takes capital, labor, energy, natural resources index, GMP, and mining environmental pollution index as internal factors, and it selects mining economic scale (MES), foreign direct investment (FDI), technology innovation (TI), and environmental regulation (ER) are used as exogenous factors [25]. GeoDetector and Tobit model are adopted to analyze internal and external factors, respectively, which can further reveal the spatial differential of mining eco-efficiency in 14 prefecture-level cities in Guangxi [66]. Based on Environmental Kuznets Theory [67], as social income increases, the eco-environment will deteriorate, which will not improve until the economy reaches a higher level. Thus, to determine the relationship between mining economic development and mining eco-efficiency, the quadratic term of mining economic scale was incorporated into the Tobit model [68].

#### 3.2.1. Analysis of Internal Factors of Eco-Efficiency of the Mining Industry: GeoDetector

The internal factor coefficients of mining eco-efficiency calculated using GeoDetector are expressed in Table 5. It shows the q statistics value of each influencing factor, where the larger the q value, the greater the degree of influence. We took 2013 as the demarcation point for the sample period due to the significant inflection and measured both the period before and after it. During 2008–2018, the influencing degree of internal factors are ranking as natural resources index (0.121) > labor (0.101) > GMP (0.067) > mining environmental pollution index (0.065) > capital (0.011). During 2008–2013, the influencing degree of internal factors are ranked as natural resources index (0.051) > capital (0.012) > GMP (0.011) > mining environmental pollution index (0.010) > labor (0.009). During 2014–2018, the influencing degree of internal factors are ranked as natural resources index (0.318) > GMP (0.164) > mining environmental pollution index (0.147) > labor (0.129) > capital (0.100). The degree of internal factors in Guangxi from 2014 to 2018 was significantly higher than that of 2008 to 2013. The ranking of factors affecting the differences in mining eco-efficiency has been rotated around 2014. Due to the low technical level of resource-based industries and high sunk costs, resource-based cities are prone to form a path-dependent progressive, which leads to a cluster of resource extraction and processing industries. Compared with other cities, resource-based cities are more likely to produce crowding effects, which caused a rebound in environmental pollution [69]. Among them, the impact of GMP and mining environmental pollution on the mining eco-efficiency has gradually increased. This phenomenon may be due to the promulgation of the “Environmental Protection Law of the People’s Republic of China” in 2014, which has promoted environmental protection to an unprecedented position, and people’s attention to environmental protection has gradually increased. Therefore, it is reflected in the significant increase in mining environmental pollution on the mining eco-efficiency. The natural resources index is the most important factor affecting the mining eco-efficiency in different periods. This further reflects that the mining development depends on a resource-based development mode. Therefore, it is necessary to transform the resource-dependent development mode and use technological innovation to fundamentally improve mining eco-efficiency in improving mining eco-efficiency and promoting the sustainable mining industry.

#### 3.2.2. Analysis of External Factors of Mining Eco-Efficiency: Panel Tobit Model

We adopted the panel Tobit regression model to verify the impact of the four external factors of MES, FDI, TI, and ER on the mining eco-efficiency. To avoid non-stationarity caused by different data dimensions in parameter estimation and to maintain the characteristics of panel data, this study adopted the natural logarithm of the relevant variable. The panel Tobit model used in this research is as follows.

Model 1:

(9) $$ME_{it}=\beta_{0}+\beta_{1}MES_{it}+\beta_{2}lnFDI_{it}+\beta_{3}TI_{it}+\beta_{4}ER_{it}+\varepsilon_{it}$$

Model 2:

(10) $$ME_{it}=\beta_{0}+\beta_{1}MES_{it}+\beta_{2}MES_{it}^{2}+\beta_{3}lnFDI_{it}+\beta_{4}TI_{it}+\beta_{5}ER_{it}+\varepsilon_{it}.$$

The Tobit model of mining eco-efficiency factors was estimated on Stata 15.0. The empirical results are presented in Table 6 below. The influencing degree of external factors are ranking as MES > TI > ER > FDI.

(1) The estimated coefficient of MES was positive and passed the 10% significance level test. For every 1% increase in MES, the mining eco-efficiency will also increase by 2.117%. This reflects that the increase in the mining economy scale will inject more capital, technology, etc., into the mining industry. A certain scale of economic investment can improve the regional mining eco-efficiency. The quadratic coefficient of the MES is positive, indicating that there is a typical “U-shaped” curve between the MES and the mining eco-efficiency, which is consistent with Wang et al. [70]. It reflects the leading role of the MES in different development stages of the mining industry. Before the turning point of the “U-shaped” curve is the early stage of mining development, which is mainly manifested as an unsustainable development mode. In the early stage of mining development, resource consumption and environmental pollution are prominent, making the mining eco-efficiency gradually decline as the MES increases. Nevertheless, with the mining economy entering a mature stage, the investment in technological innovation, the improvement of management systems, and the deepening of environmental protection concepts have gradually improved the mining eco-efficiency.

(2) The estimated coefficient of FDI was positive and passed the 1% significance level test. For every 1% increase in FDI, the mining eco-efficiency will also increase by 0.209%. The research results are consistent with Fang et al. [57]. The FDI generally has technical spillover channels by horizontal, forward, and backward, which affect the local enterprises. The FDI brings the market competition effect and demonstration imitation effect, thereby improving the technical and production efficiency of enterprises in the same industry in the host country, but the room for potential technology development is gradually narrowing. In 2008, with the advancement of the Administrative Measures on Foreign-invested Mineral Exploration Enterprises of the Ministry of Commerce and the Ministry of Land and Resources of the People’s Republic of China, the scale of the extractive industries attracting foreign investment gradually expanded, which played a positive role in the structural adjustment and green transformation of China’s mining industries.

(3) The estimated coefficient of TI was positive and passed the 10% significance level test, indicating that R&D human intensity promotes mining eco-efficiency. For every 1% increase in TI, the mining eco-efficiency will also increase by 0.419%. The traditional approach of improving mining eco-efficiency, which solely relies on structural adjustment, has a minimal effect. With the steady increase of industrial production cost, increasing mining R&D investment can fundamentally improve mining eco-efficiency and is the only way to realize continuous improvement of mining eco-efficiency.

(4) The estimated coefficient of ER has a significant positive correlation with mining eco-efficiency, which agrees with our expectation. For every 1% increase in ER, the mining eco-efficiency will also increase by 0.306%. Hence, strict environment regulation can force enterprises to reduce emissions. With the growing awareness of environmental problems in China, enterprises have no option but to choose advanced clean production technologies, facing the high cost induced by environmental regulation. In this way, enterprises will consume much less energy and emit far fewer pollutants [71].

## 4. Conclusions and Policy Implications

In this paper, mining eco-efficiency is established from the perspective of inputs and outputs, and it was split into economic efficiency, environmental efficiency, and resource efficiency. After discussing the regional difference and dynamic evolution of mining eco-efficiency, we empirically analyzed the internal and external influencing factors with GeoDectector and Tobit models, respectively. The main conclusions are as follows:

(1) The mining eco-efficiency, economic efficiency, environmental efficiency, and resource efficiency of Guangxi fluctuate considerably, mainly as follows: the economic efficiency of Guangxi has shown a downward trend; its environmental efficiency and mining efficiency have both shown a “U-shaped” curve; its resource efficiency has shown an “inverted N-shaped” curve.

(2) The economic efficiency, environmental efficiency, resource efficiency, and mining eco-efficiency have huge regional differences. Among them, cities bordering other provinces, such as Chongzuo, Beihai, Hechi, Liuzhou, Guilin, Hezhou, Wuzhou, etc., have a significantly better mining eco-efficiency than non-bordering cities.

(3) The mining eco-efficiency, economic efficiency, environmental efficiency, and resource efficiency show significant regional migration trends. The regional center has shifted to the Beibu Gulf Economic Development Zone, and the inter-regional development has become more and more balanced.

(4) In the analysis of internal influencing factors, the natural resource index has the greatest impact on the mining eco-efficiency; in the analysis of external influencing factors, the mining economic scale has the most significant impact on the mining eco-efficiency, and with the increase of the mining economic scale, the mining eco-efficiency showed a typical “U-shaped” curve.

Based on the conclusions above, we mainly put forward corresponding policy recommendations from four aspects: opening-up, technological progress, regional coordination, and government control to improve the mining eco-efficiency in Guangxi.

(1) The impact of foreign direct investment in mining eco-efficiency is very significant and positive. Related government should encourage clean extraction technologies and independent innovations conducive to clean production and energy conservation, make better use of the technological spillover effects of opening-up, and reduce product structure effects. At the same time, absorb and learn from the advanced technological achievements, management experience, and environmental protection standards of foreign-funded enterprises in the process of green industrial transformation.

(2) Schultz [72] believes that human capital is an important factor determining the regional economic growth and causing the gap between the rich and the poor. Therefore, Guangxi should always regard scientific and technological progress as the leading force in the development of the mining economy, focus on improving the quality of scientific and technological human resources, promote the effective combination of scientific and technological progress and the mining economy, play the guiding role of market and social needs, and promote the transformation of scientific and technological achievements into productive forces.

(3) The improvement of Guangxi’s mining eco-efficiency requires inter-regional cooperation and a coordinated mining development pattern. The resource-enriched area of western Guangxi should give full play to its advantages, realize the transformation of resource advantages into economic advantages, and bring advanced technology and practical experience to drive the development of mining technology and scale in the surrounding areas and the whole province. The Xijiang Economic Belt should make use of its good location advantages, actively undertake the transformation and upgrading of coastal industries, gradually accumulate funds, cultivate technology and talents, support the development of advanced mining technologies and industries, eliminate some backward industries in due course, and realize industrial development while undertaking the industrial transfer. The Beibu Gulf Economic Zone should give full play to its advantageous industries, expand its leading mining-related industries, strengthen cooperation in mining projects invested by cross-regional enterprise groups, and form a driving force for sustainable development in the future.

(4) Governments should increase the intensity of financial transfer payments in various regions, develop counterpart assistance modes, encourage developed mining economies to cooperate with underdeveloped regions, establish special development funds, and ultimately achieve mutual benefit and joint development in all regions. In addition, the government should support the merger and reorganization of mining enterprises and expand the scale of production through incentive mechanisms and corresponding supporting measures so as to improve the mining eco-efficiency. Finally, the government can strengthen the enforcement of environmental laws and regulations, improve the regional environmental protection management system and evaluation mechanism, increase environmental supervision, and strengthen the supervision of pollutant emissions from industrial enterprises. At the same time, it should introduce incentives and penalties to encourage innovation, environmental protection, and emission reduction and increase the enthusiasm of enterprises in R&D innovation and environmental protection.

## Figures and Tables

**Table 1:** Summary of DEA applications to eco-efficiency.

Reference	Research Object	Inputs	Desirable Outputs	Undesirable Outputs	Methodology
Zhang et al. [12]	Regional industrial systems’ eco-efficiency in China	Water resource Raw mining resource Energy	Value-added to industry	COD discharge Nitrogen discharge Sulfur dioxide emission Soot emission Dust emission Industrial solid wastes produced	CCR BCC
Shao et al. [29]	Eco-efficiency of China’s industrial sectors	Energy Labor Capital	Industrial value-added	CO_2_ Solid waste COD generation NH_3_-H generation SO_2_ generation Smoke dust generation	Two-stage DEA
Huang et al. [30]	Composite eco-efficiency in 30 provinces	Energy Labor Capital Water Land	GDP	Pollution index	Meta-US-SBM
Zhang et al. [22]	Industrial eco-efficiency in China	Capital, Labor Energy Environmental emissions	The gross industrial output value	--	Three-stage DEA
Wu et al. [31]	Eco-efficiency of coal-fired power plants in China	Water Oil Auxiliary power Coal Installed capacity Capital	Electricity generated Equivalent available coefficient	CO_2_ emissions Dust emission Concentration NOx emission concentration SO_2_ emission concentration	Super efficiency DEA
Yu et al. [32]	Eco-efficiency of 191 prefectural-level cities in China	Energy Labor Capital Land	GDP	Environmental pollution index	Meta-US-SBM
Masuda [33]	Eco-efficiency of wheat production in Japan	Global warming potential Aquatic eutrophication potential	Wheat yield	--	SBM-Window-DEA
Hu et al. [34]	Eco-efficiency of centralized wastewater treatment plants in 128 Chinese industrial parks	Investment Operating cost Energy Relative capacity load Wastewater	COD removal efficiency TN removal efficiency NH_3_-N removal efficiency TP removal efficiency	--	SBM-DEA
Hu and Liu [26]	Eco-efficiency in the Australian construction industry	Number of employed persons Value of construction work done	Gross value added	CO_2_ equivalent	SBM-DEA
Liu et al. [35]	Eco-efficiency of coal-fired power plants in China	Generator capacity Operation expenditure	Net generation	--	CCR Extended CCR
Zhang et al. [36]	Eco-efficiency in 102 countries	Land area Energy use Labor force	GDP	CO_2_ emissions PM2.5 emissions	Two-stage Super-SBM
Robaina-Alves et al. [37]	Eco-efficiency in 27 European countries	Energy Capital Labor	GDP	Greenhouse gas emissions	A new stochastic frontier model
Yang and Zhang [38]	Regional eco-efficiency in 30 provinces	Capital stock Labor Construction land area Water Energy	GDP	Solid waste emissions Household refuse SO_2_ emissions Soot and industrial dust emissions Waste water emissions	Global DEA

Note: Cooper–Charnes–Rhodes (CCR), Banker–Charnes–Cooper (BCC), Meta-frontier undesirable outputs super efficiency SBM (Meta-US-SBM).

**Table 2:** The selection of input indicators and output indicators.

Type		Indicator	Unit	Obs.	Min.	Max	Mean	Std. Dev.
Input	Labor	Labor force	Person	154	883	35,156	8397.92	6783.06
	Capital	Annual investment in mining	10,000 yuan	154	1686.00	4,070,893.49	65,993.92	328,381.88
	Natural resources index	Mining water consumption	100 million m^3^	154	2.02	2116.32	140.65	294.78
		Use area of the mining area	hectares	154	114.48	28,451.7	4296.26	4606.62
		Comprehensive energy consumption of mining industry	10,000 tons of SCE	154	0.10	22.06	3.43	3.46
Output	GMP	Gross mining output	10,000 yuan	154	4324.50	1,375,290.86	140,291.87	13,671.29
Undesirable output	Mining environmental pollution index	Mining wastewater discharge	10,000 tons	154	4.07	7822.18	463.16	1097.564
		Mining dust emissions	ton	154	5.037	23,210.37	876.39	2056.80
		Waste rock emissions	10,000 tons	154	0.01	675,166.00	4580.26	54,391.46

**Table 3:** The average value and ranking of the eco-efficiency by region.

Prefecture-Level Cities	Economic Efficiency	Rank	Environmental Efficiency	Rank	Resource Efficiency	Rank	Mining Eco-Efficiency	Rank
Baise	0.9052	7	0.8318	6	0.8332	8	0.7012	6
Beihai	1.1352	1	0.8683	3	1.3417	1	1.0493	1
Chongzuo	1.0242	3	0.8379	5	0.9836	2	0.9484	3
Fangchenggang	0.9238	5	0.8505	4	0.9162	4	0.6869	7
Guigang	0.7027	13	0.4916	13	0.6113	11	0.4839	10
Guilin	1.1166	2	0.9847	1	0.8969	5	1.0125	2
Hechi	0.7332	11	0.4632	14	0.6098	12	0.5769	9
Hezhou	0.7834	8	0.5038	12	0.9593	3	0.6055	8
Laibin	0.4399	14	0.5543	11	0.2606	14	0.1771	14
Liuzhou	0.7117	12	0.5827	10	0.7563	10	0.4399	13
Nanning	0.7796	9	0.6345	8	0.8236	9	0.4807	12
Qinzhou	0.7629	10	0.6254	9	0.5799	13	0.483	11
Wuzhou	0.9157	6	0.8927	2	0.8918	6	0.7109	5
Yulin	0.9399	4	0.6932	7	0.8633	7	0.7451	4

**Table 4:** Changes of elliptical parameters of standard deviation from 2008 to 2018.

	Mining Eco-Efficiency			Economic Efficiency		
Year	Center	Long and short axis ratio	Rotation	Center	Long and short axis ratio	Rotation
2008	109.12° E, 23.76° N	0.617	74.213	109.12° E, 23.58° N	0.678	70.063
2018	109.09° E, 23.07° N	1.180	92.896	109.14° E, 23.16° N	0.792	75.551
	**Environmental Efficiency**			**Resource Efficiency**		
Year	Center	Long and short axis ratio	Rotation	Center	Long and short axis ratio	Rotation
2008	108.99° E, 23.61° N	0.615	69.918	109.15° E, 23.58° N	0.609	71.736
2018	109.23° E, 23.42° N	0.752	69.335	109.09° E,22.97° N	0.871	63.816

**Table 5:** The q statistics of external factors based on GeoDetector.

Period	X1	X2	X3	X4	X5
2008–2018	0.011	0.101	0.121	0.067	0.065
2008–2013	0.012	0.009	0.051	0.011	0.010
2014–2018	0.100	0.129	0.318	0.164	0.147

Note: X1 represents Capital, X2 represents Labor, X3 represents Natural resources index, X4 represents GMP, X5 represents Mining environmental pollution index.

**Table 6:** Tobit regression results for external driving forces of mining eco-efficiency.

Variable	Model (1)	Model (2)
MES	2.117 *	−6.383
	(1.757)	(5.110)
$MES^{2}$		57.226 *
		(32.418)
lnFDI	0.209 ***	0.257 ***
	(0.047)	(0.054)
TI	0.419 *	0.419
	(0.829)	(0.819)
ER	0.360 *	0.322 *
	(0.244)	(0.243)
cons	−0.205	−0.289
	(0.239)	(0.241)

Note: ∗ and ∗∗∗ were shown to be significant at 0.1 and 0.01 levels.

## Data Availability

The data presented in this study are available on request from the corresponding author.
